# Data on the effect of knockout of cytohesin-1 in myelination-related protein kinase signaling

**DOI:** 10.1016/j.dib.2017.09.024

**Published:** 2017-09-20

**Authors:** Ruri Tsuneishi, Naoto Matsumoto, Misa Itaoka, Yuri Urai, Minami Kaneko, Natsumi Watanabe, Shou Takashima, Yoichi Seki, Takako Morimoto, Hiroyuki Sakagami, Yuki Miyamoto, Junji Yamauchi

**Affiliations:** a Laboratory of Molecular Neuroscience and Neurology, School of Life Sciences, Tokyo University of Pharmacy and Life Sciences, Hachioji, Tokyo 192-0355, Japan; b Department of Pharmacology, National Research Institute for Child Health and Development, Setagaya, Tokyo 157-8535, Japan; c Glycobiology Research Unit, The Noguchi Institute, Itabashi, Tokyo 173-0003, Japan; d Department of Anatomy, Kitasato University School of Medicine, Sagamihara, Kanagawa 252-0374, Japan

**Keywords:** Cytohesin-1, Myelination, Peripheral nervous system, Protein kinase, Signaling

## Abstract

Cytohesin-1 is the guanine-nucleotide exchange factor of Arf6, a small GTPase of Arf family, and participates in cellular morphological changes. Knockout mice of cytohesin-1 exhibit decreased myelination of neuronal axons in the peripheral nervous system (PNS) “Phosphorylation of cytohesin-1 by Fyn is required for initiation of myelination and the extent of myelination during development (Yamauchi et al., 2012) [1]”. Herein we provide the data regarding decreased phosphorylation levels of protein kinases involved in two major myelination-related kinase cascades in cytohesin-1 knockout mice.

**Specifications table**

**Table:** 

Subject area	Biology
More specific subject area	Neurobiology, molecular and cellular neuroscience, developmental biology
Type of data	Figure
How data was acquired	Immunoblotting, polymerase chain reaction
Data format	Raw data, analyzed data
Experimental factors	Protein bands are scanned and densitometrically analyzed.
Experimental features	Immunoblot, agarose gel electrophoresis photograph
Data source location	Tokyo University of Pharmacy and Life Sciences, Tokyo, Japan
Data accessibility	Data is available with this article

**Value of the data**

• The data set is of value to the scientific community to need the information for signaling molecules controlling myelination.
• The data can provide data for common intracellular signaling cascades involved in myelination.
• The data can promote further research on signaling molecules controlling myelination *in vivo*.

## 1 Data

**Fig. 1:**
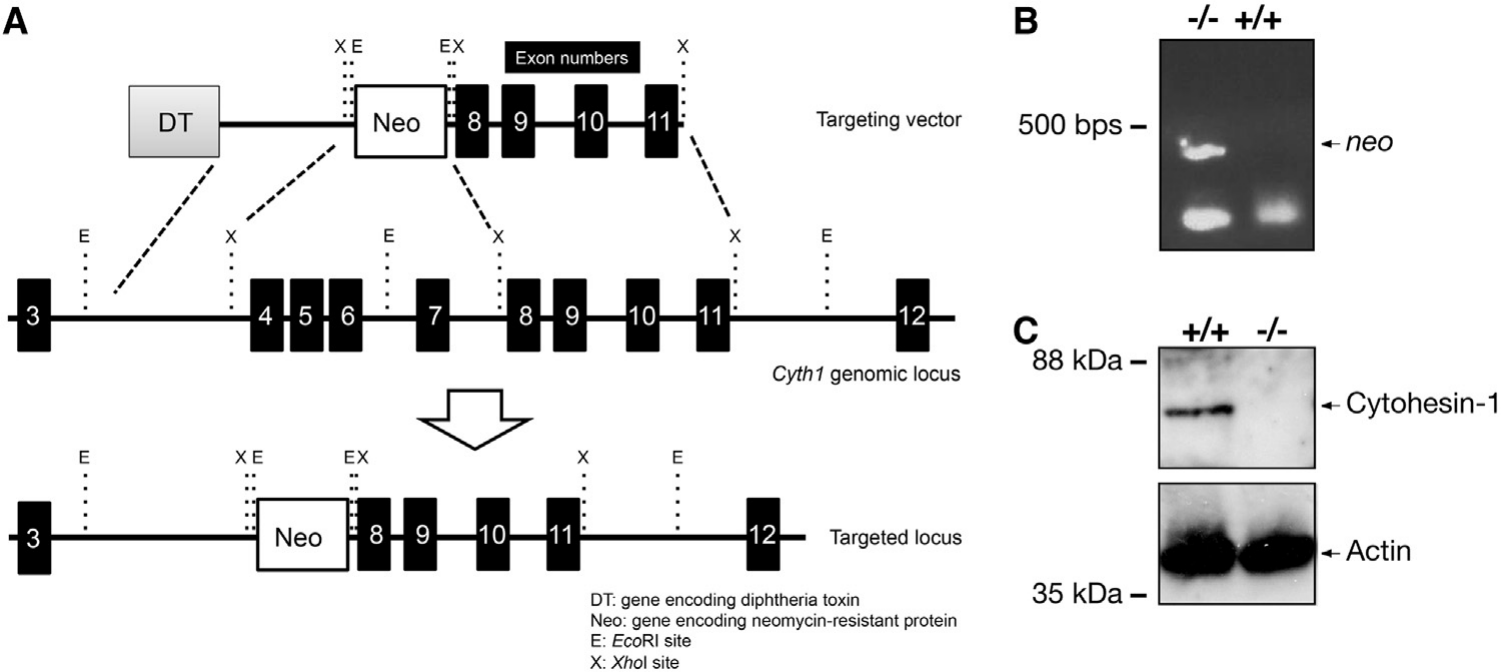
Cytohesin-1 knockout mouse. (A) Schematic strategy for generating a cytohesin-1 knockout allele. (B) Genomic PCR of cytohesin-1 knockout mouse for the *neo* gene. (C) Immunoblotting of cytohesin-1 knockout mouse sciatic nerve tissue for cytohesin-1.

**Fig. 2:**
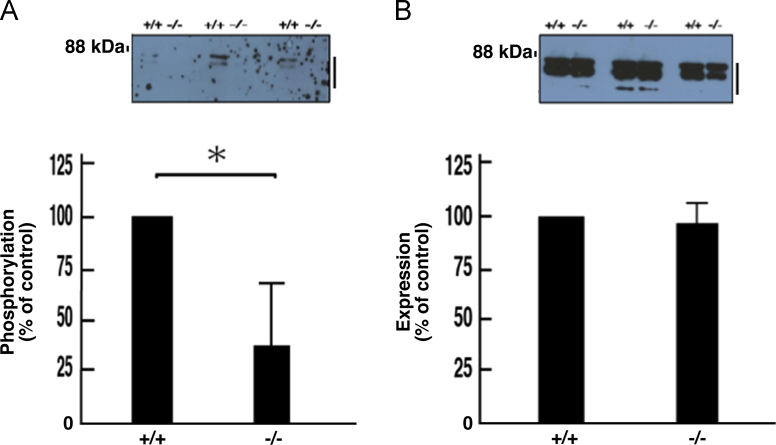
Decreased phosphorylation of Akt in cytohesin-1 knockout mice. (A) Tissue lysates (*n* = 3) from 7-day-old sciatic nerves of knockout (-/-) and control (+/+) mice were used for immunoblotting with an anti-phosphorylated Akt antibody. The scanned bands were densitometrically analyzed for quantification. (B) Tissue lysates (*n* = 3) from 7-day-old sciatic nerves of knockout (-/-) and control (+/+) mice were used for immunoblotting with an anti-Akt. The scanned bands were densitometrically analyzed for quantification. Major double bands indicate Akt1 (*top bands*) and Akt2 (*second bands*). Data were evaluated using Student's *t*-test (*, *p*< 0.01; *n* = 3).

**Fig. 3:**
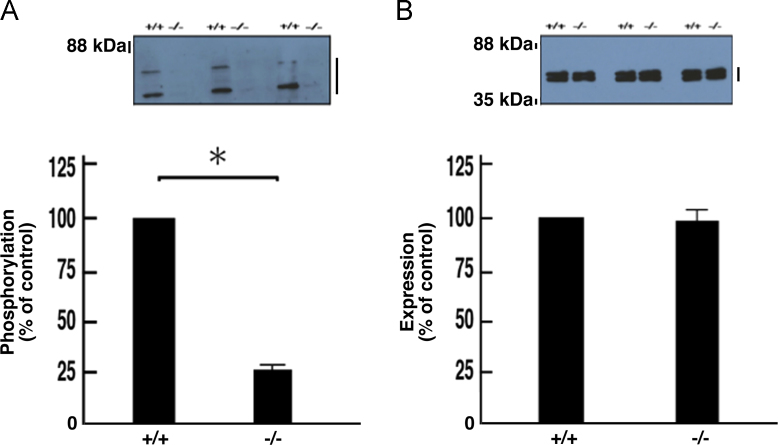
Decreased phosphorylation of ERK1/2 in cytohesin-1 knockout mice. Tissue lysates (*n* = 3) from 7-day-old sciatic nerves of knockout (-/-) and control (+/+) mice were used for immunoblotting with an anti-phosphorylated ERK1/2 (A) or anti-ERK1/2 (B) antibody. The scanned bands were densitometrically analyzed for quantification. Major double bands indicate ERK1 and ERK2. Data were evaluated using Student's *t*-test (*, *p*< 0.01; *n* = 3).

**Fig. 4:**
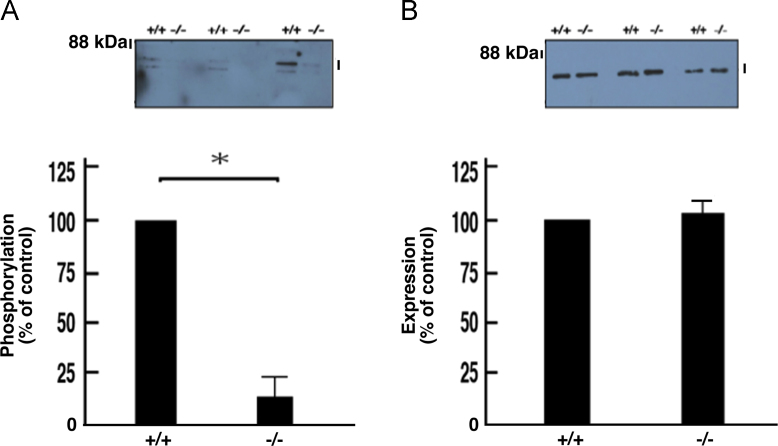
Decreased phosphorylation of MEK1/2 in cytohesin-1 knockout mice. Tissue lysates (*n* = 3) from 7-day-old sciatic nerves of knockout (-/-) and control (+/+) mice were used for immunoblotting with an anti-phosphorylated MEK1/2 (A) or anti-MEK1/2 (B) antibody. The scanned bands were densitometrically analyzed for quantification. Major bands involve MEK1 and MEK2. Data were evaluated using Student's *t*-test (*, *p*< 0.01; *n* = 3).

**Fig. 5:**
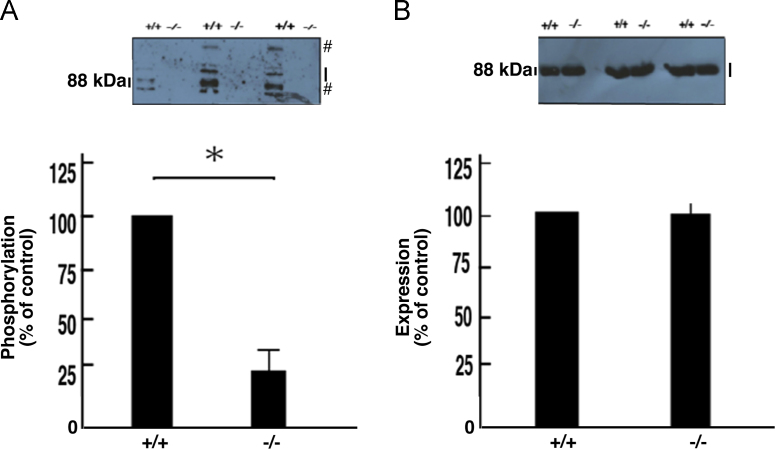
Decreased phosphorylation of B-Raf in cytohesin-1 knockout mice. Tissue lysates (*n* = 3) from 7-day-old sciatic nerves of knockout (-/-) and control (+/+) mice were used for immunoblotting with an anti-phosphorylated B-Raf (A) or anti-B-Raf (B) antibody. The scanned bands were densitometrically analyzed for quantification. Bands of approximately 88 kDa indicate B-Raf. Number signs (#) are likely to be non-specific bands. Data were evaluated using Student's *t*-test (*, *p*< 0.01; *n* = 3).

The exons 4 to 11 of the *cytohesin-1* gene were replaced with the *neo* gene (Fig. 1A). Deletion of these exons was confirmed by genomic polymerase chain reaction (PCR) and immunoblotting (Fig. 1, B and C). In immunoblotting with an antibody specific for phosphorylated Akt kinase (active Akt), decreased phosphorylation was observed in protein samples from knockout mouse nerves (Fig. 2, A and B). Akt is one of the central kinases controlling myelination [2], [3], [4], [5]. Phosphorylation of kinases belonging to the mitogen-activated protein kinase (MAPK)/extracellular signal-regulated kinase (ERK) cascade was also decreased in knockout mouse nerves (Fig. 3, Fig. 4, Fig. 5). MAPK cascade in neuronal and glial cells is composed of ERK1/2, MEK1/2, and B-Raf and is also well known to control myelination [2], [3], [4], [5].

## 2 Experimental design, materials and methods

### 2.1 Cytohesin-1 knockout mice

A 13.5-kb Xba I fragment of genomic DNA containing exons 4 to 11 of cytohesin-1 was obtained from a 129/Sv mouse genomic library. The cytohesin-1–targeting vector was constructed by replacing the ~3.6-kb Xba I fragment containing exons 4 to 7 of cytohesin-1 within the fragment containing exons 4 to 11, which was ligated to the gene encoding diphtheria toxin, with a cassette of the neomycin-resistant gene. 129/Sv embryonic stem (ES) cells were transfected with the linearized targeting vector by electroporation. These ES cells were used to generate chimeric mice. Heterozygous offspring were mated to wild-type C57BL/6JJms mice, and the mutations were propagated in this strain for at least 10 generations before it was crossed to produce homozygotes for experiments. Homozygous mice, as well as heterozygous mice, were fertile under standard breeding conditions [1]. The genomic PCR for identification of the knockout allele was performed. The primers used for genomic PCR were 5’-CCCGGTTCTTTTTGTCAAGACCGACCTGTC-3’ (sense) and 5’-CATTCGCCGCCAAGCTCTTCAGCAATATCAC-3’ (antisense) for the *neo* gene [1]. PCR amplification was performed in 30 cycles, each consisting of denaturation at 94 °C for 1 min, annealing at 68 °C for 1 min, and extension at 72 °C for 1 min. Male mice were used for experiments if it was possible to distinguish their sex.

### 2.2 Immunoblotting

Mouse sciatic nerves were lysed in lysis buffer (50 mM HEPES-NaOH, pH 7.5, 20 mM MgCl_2_, 150 mM NaCl, 1 mM dithiothreitol, 1 mM phenylmethane sulfonylfluoride, 1 μg/ml leupeptin, 1 mM EDTA, 1 mM Na_3_VO_4_, and 10 mM NaF) containing detergents (0.5% NP-40, 1% CHAPS, and 0.1% SDS) [6], [7]. The presence of these detergents is important for myelin protein isolation [6], [7]. Equal amounts of the proteins (20 μg total proteins) in centrifuged cell supernatants were heat-denatured for immunoblotting using the MiniProtean TetraElectrophoresis and TransBlot TurboTransfer System (Bio-Rad, Hercules, CA, USA). The transferred membranes were blocked with the Blocking One kit (Nacalai Tesque, Kyoto, Japan) and immunoblotted using primary antibodies, followed by peroxidase-conjugated secondary antibodies (Nacalai Tesque). The bound antibodies were detected using the ImmunoStar Zeta kit (Wako, Osaka, Japan). The scanned bands were densitometrically analyzed for quantification using UN-SCAN-IT Gel software (Silk Scientific, Orem, UT, USA). The following antibodies were used: polyclonal anti-phosphorylated pan-Akt (active, phosphorylated Ser-473), polyclonal anti-pan-Akt, polyclonal anti-phosphorylated ERK1/2 (active, phosphorylated Thr-202/Tyr-204), polyclonal anti-ERK1/2, polyclonal anti-phosphorylated MEK1/2 (active, phosphorylated Ser-218/Ser-222 for MEK1 and active, phosphorylated Ser-222/Ser-226 for MEK2), polyclonal anti-MEK1/2, polyclonal anti-phosphorylated B-Raf (active, phosphorylated Ser-445), monoclonal anti-B-Raf from Cell Signaling Technology (Danvers, MA, USA).

### 2.3 Statistical analysis

Data are presented as means ± S.D. from independent experiments. Intergroup comparisons were performed using unpaired Student's *t-*test. Differences were considered significant when *p* value was less than 0.01.
